# New insights into plasmonic hot-electron dynamics

**DOI:** 10.1038/s41377-024-01594-z

**Published:** 2024-09-10

**Authors:** Dangyuan Lei, Dong Su, Stefan A. Maier

**Affiliations:** 1 Department of Materials Science and Engineering, Center for Functional Photonics, and Hong Kong Branch of National Precious Metals Material Engineering Research Centre, City University of Hong Kong, 83 Tat Chee Avenue, Hong Kong S.A.R., 999077 China; 2 School of Physics and Astronomy, Monash University, Clayton, 3800 VIC Australia; 3 Blackett Laboratory, Imperial College London, London, SW7 2AZ UK

**Keywords:** Nanophotonics and plasmonics, Sub-wavelength optics

## Abstract

Recent advances in understanding the intricate hot-electron dynamics in plasmonic nanostructures enable efficient hot-carrier generation, transport, and manipulation, driving technological innovations in photodetection, solar cells, photocatalysis, and ultrafast nanophotonics.

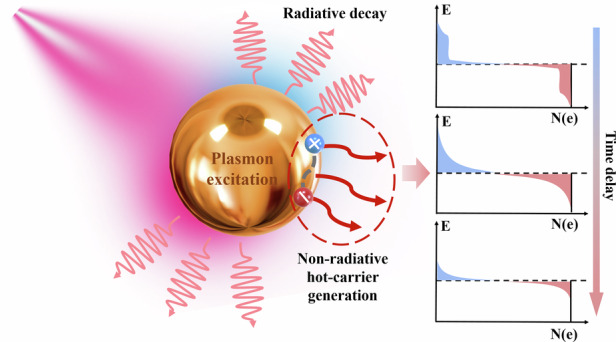

Recent advances in understanding the hot-electron dynamics in plasmonic nanostructures have significantly impacted a variety of plasmonically powered physical, chemical, and even biological processes. New insights into their quantum properties and complex relaxation mechanisms enable the efficient generation, transport, and manipulation of hot carriers, making them promising for technological applications such as wavelength-specific sub-bandgap photodetection^1,2^, plasmon-enhanced solar cells^3–5^, solar-driven photocatalysis^6–8^, and ultrafast nonlinear optical devices^9,10^.

Plasmonic excitations, typically including surface and localized plasmons in periodic and isolated metal nanostructures, efficiently couple charge density oscillations with photons, resulting in robust light absorption and transient generation of non-equilibrium hot carriers upon plasmon decay. Extraction of these carriers promptly before thermalization is possible yet difficult. Hence the effective exploitation of plasmonic hot carriers in nanophotonic devices faces challenges associated with the intricate ultrafast dynamics at the nanoscale, highlighting the need for developing deeper insights critical for understanding their optical properties and optimization of device performance^11,12^.

Khurgin et al. recently reviewed the generation and temporal evolution of hot electrons in plasmonic nanostructures, considering both microscopic and macroscopic effects from both quantum and classical perspectives^13^. In general, generating hot electrons in plasmonic structures via surface plasmon excitations offers several advantages: high generation efficiency from strong light absorption, broadband tunable properties due to the nature of plasmon resonances, and a high density of hot electrons concentrated in a well-defined volume near the metal surface. This allows for efficient extraction of hot carriers before their thermalization by engineering resonant charge injection or transfer to adjacent molecules or semiconductors^6,14^. Consequently, exploiting the advantages of plasmonically generated hot electrons could pave the way for ultrafast sub-bandgap photodetectors, nonlinear optical components, and photocatalytic devices with performance that could rival or even surpass that of traditional devices.

Localized surface plasmons (LSPs) oscillate shortly and dephase rapidly into hot charge carriers (electrons and holes), whose distribution is initially non-thermal and then evolves thermally through internal thermalization and complex external processes. This review begins with the description of hot-carrier generation and relaxation, emphasizing the discrete (quantum) nature of hot-carrier excitation in metal nanoparticles and its fundamental implications for understanding the carrier dynamics on the femtosecond scale and related nonlinear optics^13^. The discrete character of hot carriers reveals that most nanoparticles in an ensemble remain inactive under CW light illumination, with few hosting LSPs or hot carriers, whereas in the case of pulsed laser excitation, multiple LSPs on a single nanoparticle can be excited, resulting in a significant increase in the amount of generated hot carriers. Each time an LSP decays, an electron-hole pair is generated in the metal, surviving for only a very short time due to both electron-electron and electron-phonon scattering processes^15^. Leveraging femtosecond pulse excitation with even shorter pulse durations as time gates allows for deciphering the entangled relaxation mechanisms of hot carriers.

The temporal evolution of hot carriers in a plasmonic nanoparticle involves changes in the electron energy distribution before and after the decay of LSPs. This process can be divided into five stages occurring on various timescales (Fig. 1a). This review illustrates, from the quantum nature of LSPs, the ultrafast dynamics of these processes occurring in a given nanoparticle and introduces key experimental studies of the hot-carrier relaxation mechanisms. According to energy and momentum conservation laws, four mechanisms of electron-hole pair generation in nanostructured metals are compared and comprehensively explained. The direct inter-band transition is momentum-conserving, while the other three types of LSP/surface plasmon polariton (SPP) decay mechanisms, which involve momentum variation, are all intra-band transitions^16^. These intra-band transitions entail a momentum mismatch that needs to be compensated. In this regard, the decay channels due to intra-band transitions can be classified into phonon- or defect-assisted decay, electron-electron-scattering-assisted decay, and Landau damping or surface-collision-assisted decay (Fig. 1b).

**Fig. 1:**
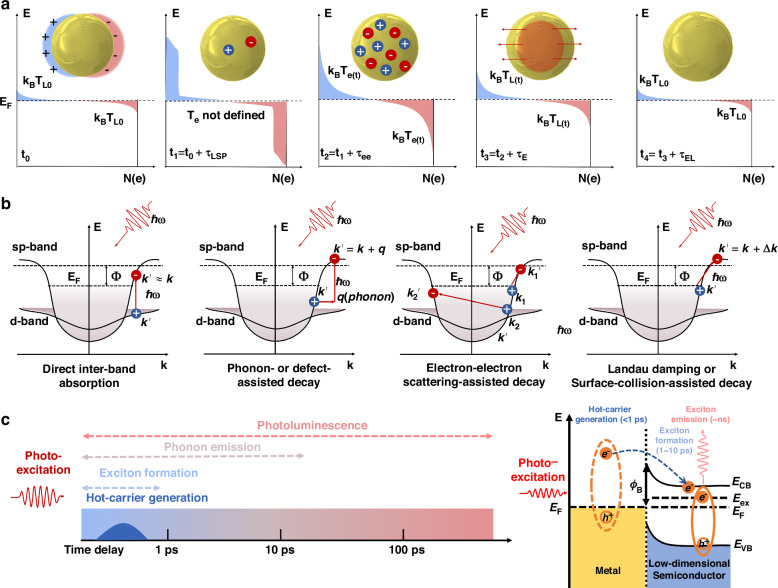
Evolution of hot carriers and relevant mechanisms of electron-hole pair generation in metal nanoparticles as well as hot-electron-mediated room-temperature generation of exciton complexes. **a** Hot-electron relaxation dynamics and energy distribution in a nanoparticle at different times. *τ*_LSP_ is the decay time of LSPs, *τ*_*ee*_ is the electron-electron scattering time, *τ*_*E*_ is the electron’s thermalization time, and *τ*_EL_ is the electron’s cooling time. *T*_*L*_ and *T*_*e*_ represent the lattice temperature and electron temperature, respectively. **b** Four mechanisms of hot-carrier generation in metals. **c** Schematic illustration of the overall relaxation processes in a hybrid metal-semiconductor low-dimensional system after optical excitation. Here the semiconductors could be layered transition metal dichalcogenides or quantum dots. **a**, **b** are adapted from ref.^13^, with modifications

Understanding the fundamental mechanisms governing hot-electron temporal behavior is crucial for advancing modern optoelectronic applications. Khurgin et al. focus on several key aspects of hot-electron behavior and its technological implications. First, they discussed hot-carrier injection at the metal-semiconductor interface, detailing how hot electrons transfer from metal to semiconductor materials while conserving energy and momentum, essentially influencing the efficiency of sub-bandgap optoelectronic devices^6,17^. Additionally, the role of hot electrons in chemical interactions is considered, highlighting their ability to trigger and control photochemical transformations with low-energy photons and potential applications in broadband photocatalysis^18,19^ and solar-driven photochemistry^20,21^. Furthermore, the nonlinear optical effects induced by hot electrons in plasmonic nanostructures are reviewed, particularly on their contribution to Kerr-type optical effects, which are vital for applications such as ultrafast detectors and nanoscale temperature control^22^. This timely review offers a comprehensive exploration of this promising research area, providing an excellent overview for enhancing our understanding of hot-electron behavior in plasmonic nanostructures and their implications for future technologies.

Beyond the scope of the present review, it is noteworthy that the integration of plasmonic nanostructures with low-dimensional semiconductors, including 0D quantum dots and 2D transition metal dichalcogenides, brings even more exciting opportunities for hot-carrier science and technology. For example, hot electrons can facilitate the room-temperature formation of exciton complexes, thereby constituting a many-body system at ambient conditions, such as charged bi-excitons (comprising three electrons and two holes through Coulomb interaction)^23,24^, which would otherwise be impossible due to their binding energies substantially smaller than the room-temperature thermal energy (about 25 meV). As illustrated in Fig. 1c, this unconventional phenomenon can now be realized through the rapid injection of hot electrons from a metal nanoparticle into an adjacent 2D semiconductor. Under either CW or pulsed laser pumping, hot carriers can be generated within an extremely short duration of less than 1 ps. This process occurs slightly faster than the formation of 2D excitons and significantly faster than both their non-radiative (phonon emission, more than 10 ps) and radiative relaxation processes (photoluminescence, with lifetimes roughly from 100 ps to 1 ns). Consequently, these hot electrons possess the ability to effectively modulate the formation of exciton states and the associated radiation processes. On the other hand, such ultrafast injection of hot electrons into 2D TMDCs and their inhomogeneous spatial distribution could induce a transient symmetry breaking of the crystal structure and hence invoke originally symmetry-forbidden nonlinear processes such as out-of-plane second-harmonic generation^25,26^. Finally, it is also noted that LSPs in resonant plasmonic nanostructures can scatter into free-space photons through radiative decay and hence reduce the absolute quantum efficiency of hot-carrier generation^27,28^, highlighting the importance of rational designs of plasmonic nanostructures by considering the influence of their size, shape, composition, and energy levels.

In conclusion, the transient generation, transport, and control of hot electrons in plasmonic nanostructures present unparalleled opportunities for nanophotonics and hybrid low-dimensional systems. Key unresolved issues highlight the complexity of this multi-interdisciplinary field. Understanding relevant quantum processes is crucial due to the discrete nature of light interaction with plasmonic modes, affecting hot-carrier dynamics under varying illumination conditions. On the one hand, material properties significantly influence the non-equilibrium dynamics of hot-carrier excitation and decay, demanding further exploration of electron-electron, electron-phonon, and electron-structural-defect interactions. Additionally, enhancing the nonlinear response through precise manipulation of hot-carrier dynamics remains challenging, necessitating advancements in both materials’ science and the utilization of nanostructured media^29^. Perhaps, an alternative approach to decipher the entangled hot-carrier physics is to bridge the two seemingly separate but closely connected research areas of molecular electronics and nanoscale plasmonics^30–34^, which could provide a unique multidimensional perspective on the optical and charge transport characteristics in plasmonic molecular junctions. This hybrid approach will not only offer a fundamental understanding of light-matter interactions and quantum transport at the atomic and molecular scale^35^, but also hold great promise for the development of molecular-scale optical and electronic devices. On the other hand, improved theoretical models are also essential for accurately describing non-equilibrium processes in hybrid molecular-plasmonic structures, including electron gas behavior and inter-component electron transfer mechanisms. Addressing these challenges has the potential to leverage our understanding and utilization of hot-electron dynamics in plasmonic nanostructures, paving the way for advancements in sensing, energy conversion, and advanced photonic technologies.
